# Novel swing-assist un-motorized exoskeletons for gait training

**DOI:** 10.1186/1743-0003-6-24

**Published:** 2009-07-03

**Authors:** Kalyan K Mankala, Sai K Banala, Sunil K Agrawal

**Affiliations:** 1 Department of Mechanical Engineering, University of Delaware, Newark, DE 19716, USA

## Abstract

**Background:**

Robotics is emerging as a promising tool for functional training of human movement. Much of the research in this area over the last decade has focused on upper extremity orthotic devices. Some recent commercial designs proposed for the lower extremity are powered and expensive – hence, these could have limited affordability by most clinics. In this paper, we present a novel un-motorized bilateral exoskeleton that can be used to assist in treadmill training of motor-impaired patients, such as with motor-incomplete spinal cord injury. The exoskeleton is designed such that the human leg will have a desirable swing motion, once it is strapped to the exoskeleton. Since this exoskeleton is un-motorized, it can potentially be produced cheaply and could reduce the physical demand on therapists during treadmill training.

**Results:**

A swing-assist bilateral exoskeleton was designed and fabricated at the University of Delaware having the following salient features: (i) The design uses torsional springs at the hip and the knee joints to assist the swing motion. The springs get charged by the treadmill during stance phase of the leg and provide propulsion forces to the leg during swing. (ii) The design of the exoskeleton uses simple dynamic models of sagittal plane walking, which are used to optimize the parameters of the springs so that the foot can clear the ground and have a desirable forward motion during walking. The bilateral exoskeleton was tested on a healthy subject during treadmill walking for a range of walking speeds between 1.0 mph and 4.0 mph. Joint encoders and interface force-torque sensors mounted on the exoskeleton were used to evaluate the effectiveness of the exoskeleton in terms of the hip and knee joint torques applied by the human during treadmill walking.

**Conclusion:**

We compared two different cases. In case 1, we estimated the torque applied by the human joints when walking with the device using the joint kinematic data and interface force-torque sensors. In case 2, we calculated the required torque to perform a similar gait only using the kinematic data collected from joint motion sensors. On analysis, we found that at 2.0 mph, the device was effective in reducing the maximum hip torque requirement and the knee joint torque during the beginning of the swing. These behaviors were retained as the treadmill speed was changed between 1–4 mph. These results were remarkable considering the simplicity of the dynamic model, model uncertainty, non-ideal spring behavior, and friction in the joints. We believe that the results can be further improved in the future. Nevertheless, this promises to provide a useful and effective methodolgy for design of un-motorized exoskeletons to assist and train swing of motor-impaired patients.

## Background

The incidence of spinal cord injury (SCI)in the United States is approximately 11,000 per year, with a prevalence of nearly 250,000 [1]. Damage to the spinal cord often impacts walking functions. Approximately, 52% of this population has motor incomplete lesions [1], therefore, the potential to regain functional ambulation. Rehabilitation targets restoring these functions. Currently, therapist assisted body-weight supported treadmill training (BWSTT) is used for such patient groups. In this training, a patient walks on a motorized treadmill with a harness that partially unloads the weight of the trunk from the supporting leg, while therapists help the patient in moving the legs and trunk manually [2-4]. Clinical trials with BWSTT in iSCI patients show that it is safe and results in improvements in walking[5,6]. Despite these benefits, clinical practice of BWSTT is limited because a number of therapists are required to manually facilitate the step training [3,7]. The duration of such a training is often limited by the rapist fatigue.

MIME, ARM and MIT-MANUS represent early advances in robotic devices for use in upper extremity training and rehabilitation [8-10]. These devices, and a majority of newer rehabilitation machines for the upper extremity, are powered. A second group of upper extremity machines is un-motorized or passive. This group consists of gravity balancing orthoses, which are designed for people with limited strength [11-14]. These un-motorized machines provide benefits similar to motorized machines, in a restricted way, but do not require sophisticated electronics or power sources to run the machine. As a result, they can be more affordable and possibly require less oversight by trained engineering personnel in future.

Lower extremity machines are emerging in recent years for gait training, but they are still not common in rehabilitation clinics. The design of lower extremity machines is more involved compared to those for the upper extremity because issues of posture, balance, and limb movement need to be simulatneously addressed within the design. Lokomat is a motorized bilateral exoskeleton for hip and knee joints, designed for spinal cord injury patients to be used on a treadmill [15]. Mechanized Gait Trainer (MGT) is a single degree-of-freedom powered machine that drives a foot using a crank and rocker system (Hesse and Uhlenbrock, 2000). An active leg exoskeleton (ALEX) was recently developed at the University of Delaware by the author's group which was shown to successfully alter the gait of a healthy and stroke subjects walking on a treadmill [16,17].

Using Lokomat with body weight support, Hornby et al [18] and others have shown that significant improvements can be achieved in walking of patients with chronic and sub-acute SCI. However, the cost of such a device runs in several hundreds of thousands US $, which make these prohibitive for many rehabilitation facilities and unaffordable by hospitals in under-developed countries. To increase the accessibility and success of BWSTT, costs of the therapy should be minimized.

Gottschall and Kram [19] suggested simple, non-motorized, devices which can apply forces to assist the limb swing and propel the leg foward during walking. They applied forces using rubber bands at the foot or pelvis by a spring-loaded pulley system. Even though their swing-assist devices need further developments, their results suggest that simple devices can assist those with reduced voluntary force production, such as subjects with iSCI. The non-motorized lower extremity gravity balancing orthosis (GBO), that eliminates or reduces the effects of gravity on the joints, have been used for training studies on chronic stroke patient and yielded favorable results by the author's group [20-22].

However, the design of GBO is fundamentally different from the design philosophy of the swing-assist exoskeleton presented in this paper, as the latter is motivated from providing propulsive forces to the leg during walking. We believe that the design presented in this paper is unique since it presents a simple un-motorized bilateral exoskeleton for swing assistance. In order to scientifically design the orthosis, we use the dynamics of walking to predict and optimize the motion of a leg, once it is strapped into an orthosis. The model of the swing leg provides a framework for optimization of the parameters of the exoskeleton, which are torsion springs at the hip and the knee joint.

The organization of the paper is as follows: In Section, we describe the dynamics of the human leg during swing and provide a framework for optimizing the parameters of the exoskeleton to obtain a feasible gait. In Section, we discuss the physical design of the exoskeleton and its interface with a human subject during treadmill walking. The analysis of the data collected during treadmill walking and their interpretations are also discussed. These are followed by conclusions of the work.

## Methods

### Sagittal Plane Model of Human Walking

**Figure 1:**
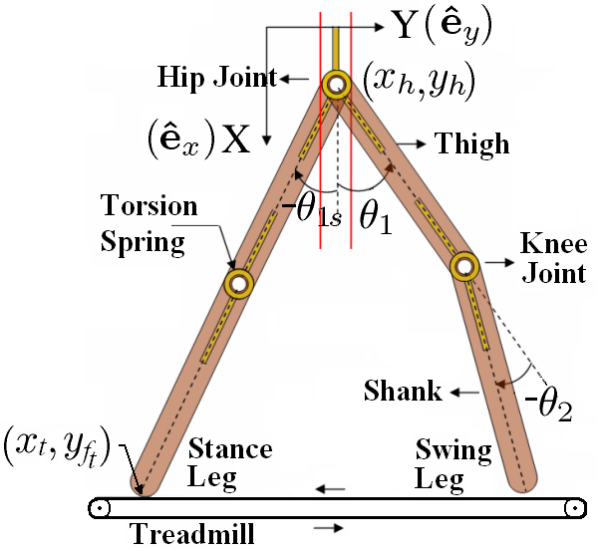
**Model schematic**. Model of a human leg in the sagittal plane with hip moving as an inverted pendulum.

Figure 1 shows the model of a human leg moving on a treadmill in the sagittal plane(X-Y plane). The Leg is modeled as having two links – thigh, shank and two joints – hip and knee. The foot is considered as a point mass at the end of shank segment (i.e., at ankle joint). The swing assistance device consists of two torsion springs – one at the hip joint and the other at the knee joint. The stiffness constants *c*_1_, *c*_2 _and the equilibrium configurations ,  of these springs are considered to be design parameters.

The system dynamics depends on the following quantities: *m*_1_, *m*_2 _– masses of the thigh and shank (leg + device); *L*_1_, *L*_2 _– lengths of thigh and shank segments; ,  – location of the center of mass of the thigh and shank (leg + device) measured from their respective joints; *I*_1_, *I*_2 _– inertia of thigh and shank (leg + device) about their center of mass. Please note that '(leg+ device)' indicates the equivalent quantity based on human leg and device parameters. *Simulation results *section shows how the equivalent parameters are calculated based on anthropometric data and device mass assumptions.

In our study, we have used two different models for the hip motion: (i) hip is inertially fixed, (ii) hip has only vertical motion, i.e., it is assumed to remain fixed in the horizontal direction. While more complex models could have been made to describe the human hip motion, we believe that pendular motion of the hip in the sagittal plane may be a reasonable first model. A spinal cord injury patient, by himself or herself, has very little residual motion left in the limbs and the sagittal plane motion will be the predominant motion during their treadmill training. In this paper, we only describe the second model, where the hip has only vertical motion (represented by red lines in Figure 1). We believe that this model is more realistic to capture the movement on a treadmill.

In this model, we assume that the foot of the stance leg remains in contact with the treadmill and moves along with it until the swing leg makes contact with the treadmill again. We also assume that the knee in the stance leg remains locked. With these assumptions, using the kinematic model of the stance leg, we compute the up and down motion of the hip. This motion is then used in the dynamics of the swing leg.

#### Hip Motion

If the treadmill moves at a constant speed *v*, the position of the contact point of the stance leg with the treadmill, *Y*_*ft *_at time *t*, is given as

(1)

where  is the position of the contact point at the start of the stance phase. Let *x*_*t *_be the position of treadmill in the  direction. Using kinematics, we write the vertical position of the hip as

(2)

(3)

Hip angle during stance phase *θ*_1*s *_is given as

(4)

#### Equations of Motion

Swing leg dynamics can be written using the Lagrange equations.

(5)

where *τ*_*i *_denotes the external torque applied at the joints. The Lagrange function ℒ given in the above equation is defined as

(6)

Where

(7)

(8)

(9)

(10)

In the above equation,  and  are unit vectors along X and Y axes.

Note that while finding the device parameters from simulations we assume that the external torque *τ*_*i *_applied is zero and based on the above dynamics we find *θ*_*i*_(*t*). Whereas while analyzing the experimental results, based on the encoders data we know *θ*_*i*_(*t*). We use this information to calculate the external torque *τ*_*i*_, more specifically the human applied component. In the later case, external torque *τ*_*i *_can be treated as a summation of device interface torques *τ*_*FT *_(which is known as it is recorded by Force-Torque (F/T) sensors) and the human applied torque *τ*_*h*_. Based on the dynamic equations we can estimate human applied torque *τ*_*h*_.

#### Knee Lock and Unlock

In human walking, the knee joint does not allow the shank to move past *θ*_2 _= 0. This locking of the joint is an instantaneous knee impact event. We account for the knee locking and unlocking during our simulations. Once the knee locks, the number of dynamic equations in (5) changes from 2 to1. During the phase of locking, as typically done during modeling of impact, the angles are considered to be continuous while the rates have an instantaneous jump. The new joint rate for the hip is computed by angular momentum conservation about the hip joint.

(11)

(12)

In the above equations '+' indicates quantities after impact and '-' indicates before impact. *H*_*O*, *leg *_denotes the angular momentum of the leg about the hip joint, *L*_*c *_denotes the location of center of mass of the whole leg (assuming it is straight, which it is after impact (knee locking)) from the hip joint, *m *denotes the mass of the whole leg (*m*_1 _+*m*_2_), *I *denotes the moment of inertia of the whole leg about its center of mass. Equating the angular momentum before and after impact, we obtain  from the knowledge of *θ*_1_, *θ*_2_,  and . Please note that *ω *in Eq. (7) and  in the above equations refer to the same quantity. After locking, the thigh and shank segments rotate about the hip joint as a single link. Knee unlocks when the equation for reaction torque at knee joint is not positive. Reaction torque is positive when knee is locked and does not exist(becomes zero) when the knee unlocks. Hence, the equation for the reaction torque has a zero crossing (value changes from being positive to negative) at unlocking event. This condition is expressed as

(13)

In the above equation, the first term represents reaction torue due to gravity, the second term represents reaction torque due to torsion spring and the third represents reaction torque due to shank acceleration. Based on day to day observations of healthy subjects walking on a treadmill it is observed that the knee does not unlock until the swing leg touches the ground.

#### Design Optimization

**Figure 2:**
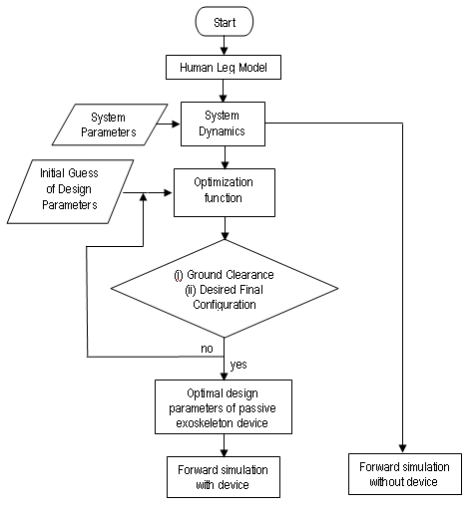
**Design optimization**. Schematic of device parameter optimization process used in the design of the swing assistive orthosis. As a first step, System Dynamics are obtained for a particular model of Human leg motion. Using the dynamics, optimization is carried out to find out device parameters. Error from desired final configuration is taken as objective function. Positive ground clearance at discrete points is taken as a constraint. Comparision is made in simuations with and without passive device before building the hardware.

The optimization of the design is schematically described in Figure 2. Given the desired initial and final configurations of the swing leg, the design parameters *c*_1_, *c*_2_, ,  are found from an optimization routine that gives a feasible gait. During optimization, the system dynamic equations were used to predict the gait. Inclusion of locking and unlocking (impact) events in dynamics would introduce discontinuities in states and increase the time of integration due to the inherent need to detect these events. These would typically slow down the optimization solution convergence. In order to speed up the integration of dynamics, during optimization, knee locking was approximated with an additional stiff spring that applies torque only when the knee angle *θ*_2 _> 0. The use of stiff spring simplified the numerical integration and helped converge to a solution faster.

Error from the desired final configuration (not the entire gait) was taken as the objective function that the optimization process would minimize. In addition, positive ground clearance (the relative (vertical) position of the foot w.r.t. the treadmill is greater than zero) at a finite number of points during the gait was imposed as a constraint. The optimized parameters were then used to perform forward simulations of the leg. During these forward simulations, locking event was not simplified with the stiff spring but instead the exact model described in *knee lock and unlock *section was used. Actual values of the desired starting and final configurations are given in the *simulations results *section.

#### Simulation Results

Device parameters are found based on the following healthy subject's biological data on whom the experimental tests were also conducted.

BodyWt = 72.6 kg

Height = 167 cm

Age = 35 yrs

*L*_thigh _= 0.41 m

*L*_shank _= 0.40 m

The following average anthropometric data for human leg [23] was used to obtain the other important parameters required for simulations.

*m*_thigh _= 0.1000 × Body Wt

*m*_shank _= 0.0465 × Body Wt

*m*_foot _= 0.0145 × Body Wt = foot mass

 = 0.433 × *L*_thigh _(center of mass of thigh from hip joint)

 = 0.433 × *L*_shank _(center of mass of shank from hip joint)

*R*_thigh _= 0.323 × *L*_thigh _(radius of gyration of thigh)

*R*_shank _= 0.302 × *L*_shank _(radius of gyration of shank)

Apart from the thigh and shank mass, in this simulation, we also considered foot mass and device mass. We assumed that the mass of the thigh and shank segments of the deviceis 1 kg each and is distributed such that their center of mass and radius of gyration coincide with center of mass and radius of gyration of human thigh and shank segments repectively. Based on the anthopometric data and the device mass assumptions, the equivalent mass and center of mass parameters to be used in the simulation can be found as follows,

*m*_1 _= *m*_thigh _+ *m*_device_thigh_

*m*_2 _= *m*_shank _+ *m*_foot _+ *m*_device_shank_

*L*_1 _= *L*_thigh_

*L*_2 _= *L*_shank_

 = 

 = ((*m*_shank _+ *m*_device_shank_) *  + *m*_foot _* *L*_2_)/(*m*_2_)

The initial configuration of the swing leg was selected as  and the final desired configuration . These configurations are chosen based on normal human gait data. Desired swing time (*t*_des_swing_) was chosen as 0.7 s. As the velocity of the hip joint at the beginning of swing phase is related (equal) to the velocity of the hip joint at the end of the stance phase, the intial velocity of hip joint can be calculated as follows,

(14)

where, *v *is the treadmill velocity which can be calculated from the kinematics and the desired swing time specification as follows,

(15)

For the stance leg, we specify the symmetrically opposite initial conditions, i.e., the final configuration of swing leg is taken as the initial configuration of the stance leg and vice versa. With these system parameters and desired configurations, the optimization routine gives the design parameters as *c*_1 _= 7.9 Nm/rad, *c*_2 _= 5.3 Nm/rad,  = 22°,  = 0°.

**Figure 3:**
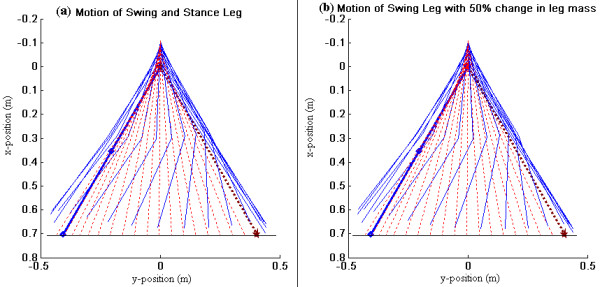
**Simulation result with device**. Motion of stance leg and swing leg – (i) with assistive device and optimal parameters of the torsional spring; (ii) With assistive device and optimal parameters of the torsional spring but with 50% change in leg mass. Stance leg – red dotted line. Swing leg – blue solid line. Initial position of swing leg – thick blue line with diamond markers. Final position of swing leg – brown line with star makers.

Using these optimized design parameters, we performed one step and multistep simulations. Figure 3 shows the stick diagrams of leg motion for one step simulation. The red dotted line shows the motion of stance leg and the blue solid line shows the motion of swing leg. The initial position of swing leg is shown by a thick blue line with diamond markers and the desired final position is shown by a brown line with star markers. Figure 3(i) shows the leg motion when the device is used with optimized design parameters – swing leg has good ground clearance and goes close to the desired final configuration. Figure 3(ii) shows the leg motion when the design parameters are kept constant but the leg mass is changed by 50% – even in this case swing leg reaches goal point in a desirable manner. The gait in these cases takes between 0.8 and 0.85 seconds to complete (which corresponds to a treadmill speed of around 2 mph). These results show that the system is robust to variations in leg mass.

**Figure 4:**
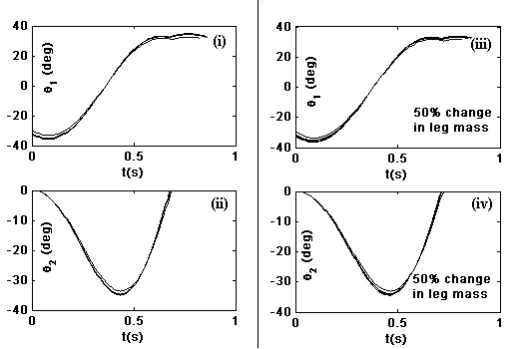
**Joint trajectories for 100 step simulation**. Joint trajectories of swing leg for 100 step simulation with optimial parameters of torsion spring – (i) *θ*_1 _vs time (ii) *θ*_2 _vs time. With 50% change in leg mass – (iii) *θ*_1 _vs time (iv) *θ*_2 _vs time

For a multi step simulation, we use the configuration of leg from previous step as a initial configuration for the next step. Figure 4 shows the joint trajectories of the swing leg for a 100 step simulation. We see that the joint trajectories are almost same during the 100 step simulation, suggesting that the trajectory is stable and also robust to changes in leg mass. In *θ*_2 _plots, we see that when *θ*_2 _reaches 0 degrees and stays at zero (i.e., the joint velocity abruptly changes to zero). This is due to the knee locking event. The joint velocity continues to be zero until the leg touches the treadmill suggesting that the knee unlocking event is not taking place.

## Discussion

Our results from the simulation resulted in a more natural human walking under the condition when the hip was allowed to move up and down, compared to the case when the hip remains inertially fixed. This is consistent with human walking, where the hip moves up and down. From the perspective of energy flow, the springs get charged during the stance phase by the treadmill and the body-weight support system which allows only a vertical motion to the hip. In swing phase, the potential energy stored in springs is converted to kinetic energy of the swing leg. Some energy flows out at the hip, working against the constraint of only vertical motion, and some energy is lost during knee and heel impact. In human walking, there is a finite-time when the leg is in double support. In this phase, both swing and stance legs are in contact with the ground. In future, if the foot is modeled as a separate limb, this double support phase of human walking can also be accounted.

### Experimental Results and Discussion

#### Exoskeleton Design

**Figure 5:**
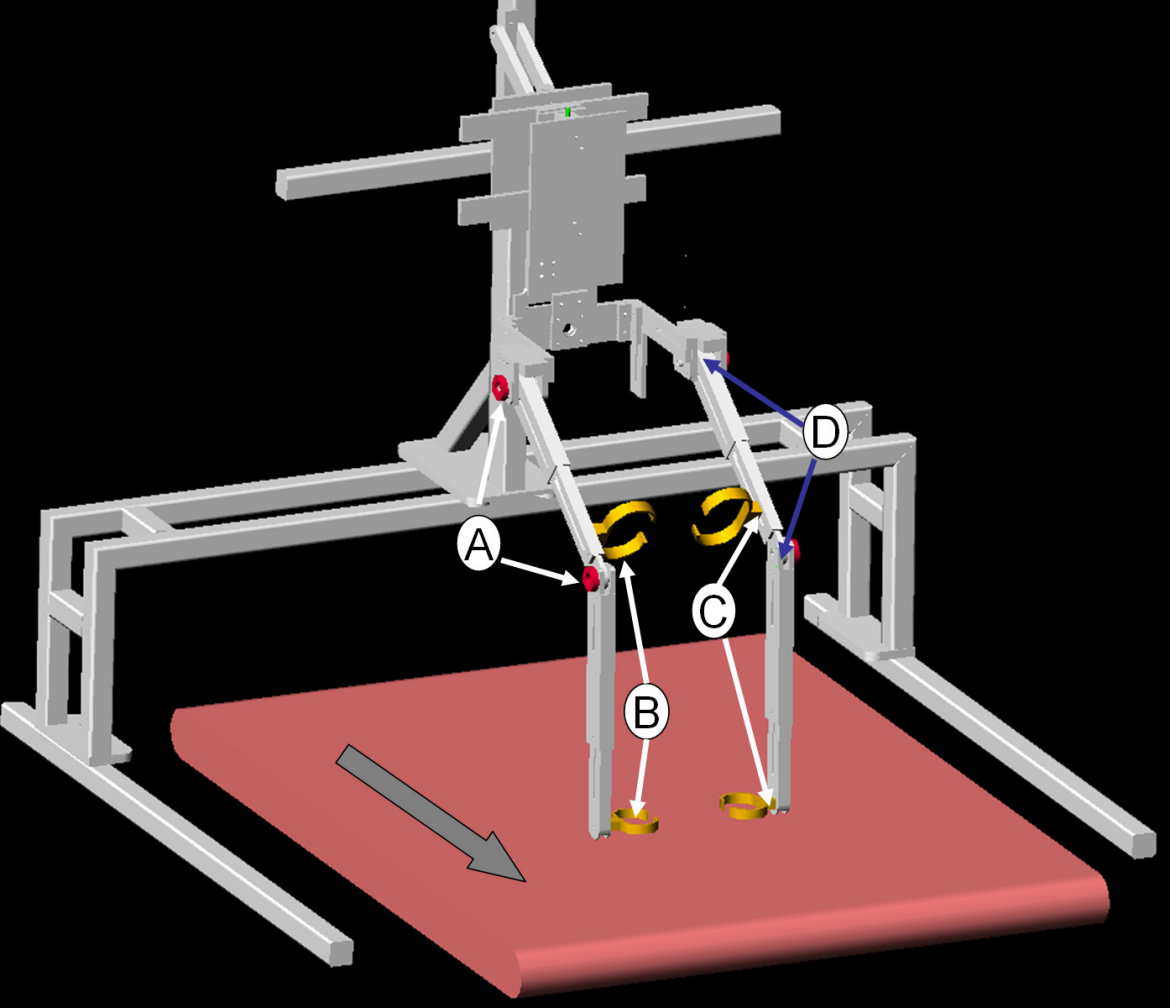
**Device drawing in AutoCAD**. AutoCAD drawing of Swing Assistance Device with Body Weight Support system and treadmill – A. Torque Springs B. Straps C. Force Torque Sensors at robot human interface D. Encoders at the Joints.

**Figure 6:**
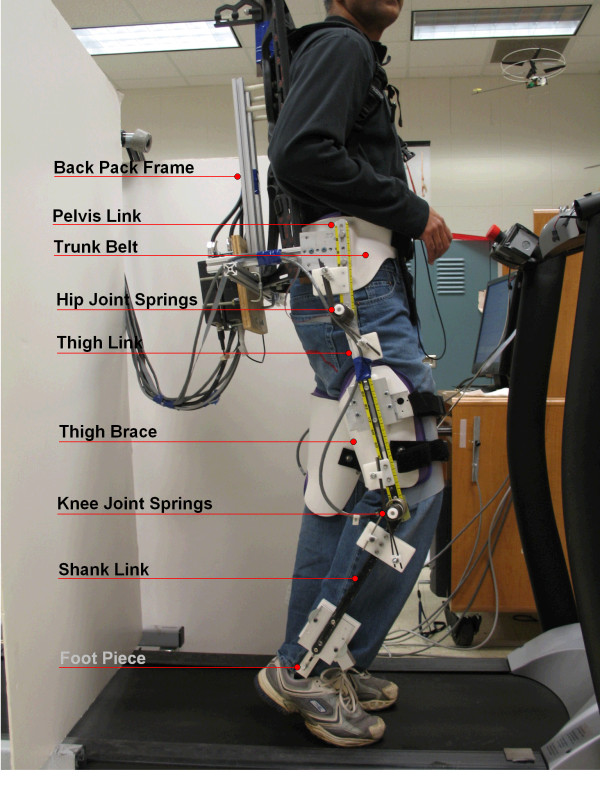
**Experimental setup**. A healthy subject wearing the swing assist exoskeleton while standing on a treadmill.

Figure 5 shows an AutoCAD drawing of an exoskeleton that was built using this design philosophy. This AutoCAD drawing lists the various components, including the adjustable limb segments to accomodate a range of subjects, the bracing attachments for the leg, the back support system that allows the trunk to move up and down, the force-torque sensors to compute the human applied joint torques, and the swing assistive torsional springs at the joints. Figure 6 shows the fabricated exoskeleton worn by a healthy subject. The device has a belt that straps onto the human trunk. Please note that this fabricated exoskeleton does not support the weight of the human subject.

A pelvic link made of aluminum is attached rigidly to the trunk belt. In order to help the pelvis remain nearly vertical during treadmill walking, a back pack frame is used. This back pack frame is rigidly connected to the pelvic link through aluminum sections. Other links in the device are the telescopic thigh and shank segments, connected successively through revolute joints. All links have slots to adjust the link lengths and match these to the human wearing it. The device thigh is connected to the human thigh with the help of a thigh brace. The device shank is connected to the human foot via a foot piece. Currently, the foot piece only allows sagittal plane ankle motion. At the device hip and knee joints, torsion springs are connected in parallel to obtain a desired stiffness and equilibrium configuration, suggested by the optimization. Encoders are mounted at all revolute joints to measure hip and knee angles. Two force-torque sensors are mounted on each leg of the exoskeleton, one sandwiched between the thigh link and the thigh brace and the other between the shank link and the foot piece. These sensors measure the forces and torques transmitted between the device and the human.

#### Data Collection

The exoskeleton was first adjusted to match the limb lengths of the subject, a 45 years healthy male of Asian origin, 70 inches tall. The subject's biological data was used to find the optimal spring parameters while walking on the treadmill at a speed of around 2.0 mph (see *simulation results *section). The appropriate springs were mounted on the exoskeleton. Note that in a clinical setting too, based on test subjects' biological data, device parameters can be found from the simulations. Once the desired stiffness parameters are obtained, the device joints' stiffness can be approximately adjusted based on an existing collection of springs. The equilibrium configurations of the springs can then be suitably adjusted if the parts used to mount the springs have slots or set of holes instead of a single hole that would allow only a single equilibrium configuration.

**Figure 7:**
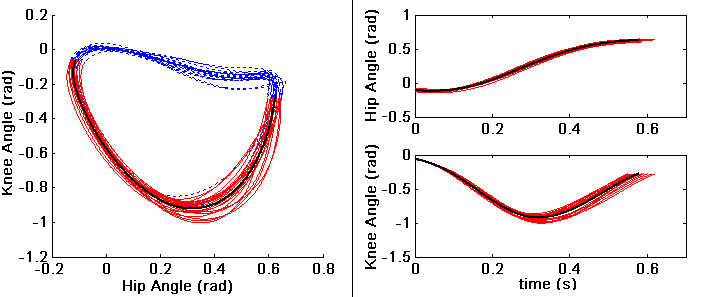
**Joint trajectories from an experimetal result**. (a) Hip versus Knee during a trial when treadmill speed was 2 mph. Red lines represent swing phase extracted from full step data represented by red and blue lines combined. Solid black lines represent average swing phase. (b) Hip angle vs time (c) Knee angle vs time.

In the current device, the encoder and force-torque sensor data were collected using a dSpace 1103 system at 1000 Hz. The force-torque sensors were manufactured by ATI and the encoders by USDigital. The subject walked on the treadmill for 15 minutes with the exoskeleton to become acclimated. Data was collected when a subject walked on a treadmill at different speeds, ranging from 1.0 mph to 4.0 mph. Figure 7(a) shows the joint data, *θ*_2 _vs *θ*_1_, of a trial where the treadmill speed was 2 mph. Note that the design was optimized for walking at a treadmill speed of around 2.0 mph; hence, we show the results of this trial in more detail. In this figure, multiple loops indicate multiple steps during a trial. Red lines represent just the swing phase, extracted from the full step data represented by both red and blue lines. Solid black line represents the average swing data, computed by averaging over the multiple cycles. In order to perform averaging, we normalized the step data to a fixed time length. The same data is plotted against time in Figs. 7(b), (c). A 20 point moving average was used to smoothen the joint encoder data to compute the joint velocity and acceleration, using central difference scheme.

#### Data Interpretation

**Figure 8:**
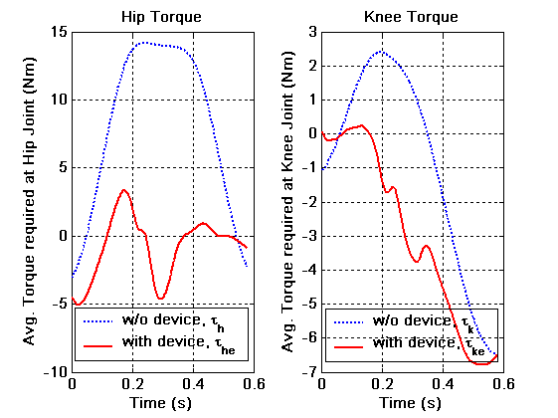
**Joint torques corresponding to the experimental result**. Estimate of torque applied by the subject at the hip and the knee joints for a treadmill speed of 2.0 mph. Blue – with the exoskeleton, Red – without the exoskeleton.

We analyzed the data using two methods to study the performance differences with and without the spring assist: (i) We estimated the joint torques applied by the human during swing using the kinematic data obtained from the joint encoders and the force-torque data obtained from the interface force-torque sensors in conjunction with the leg dynamics given in Sec. (ii) We estimated the human applied joint torque using the dynamic model, where the inputs to this model are the kinematics recorded by the joint sensors. The second approach does not use the interface force-torque data in the computations and hence represents the torque needed to excute the same trajectory as in case (i) but without the spring assist. In an ideal situation, if the exoskeleton was working completely according to the intended design, one would expect to see that the joint torques in (i) are closer to zero, or much less compared to those predicted in (ii). For the kinematic data shown in Figure 7, the torques required by the human in the two cases are shown in Figure 8. In these plots, solid red lines correspond to (i), while the dotted blue lines to (ii). Ideally, as we mentioned earlier, one would expect to see the joint torques required by human to be smaller in the device, since the device parameters were found based on the assumption of zero-input from human. In Figure 8, we see that the magnitude of the hip joint torque in (i) is smaller – peak torques bounded by (≈5 Nm) compared to (≈14.5 Nm) in (ii) – indicating that a subject with less than normal muscle strength maybe able toper form this gait while wearing the device. A similar comparison for knee joint torque shows that the absolute torque with the device is favorable during the early part of the swing but becomes comparable to the magnitude of the torque without it during the later part of the swing. These results indicate that the exoskeleton performs favorably over the swing at the designed treadmill speed, since it reduces the magnitude of the hip and knee joint torque. However, there is still room for improvement in performance of the exoskeleton. These results are remarkable considering the following observations:(i) the design is based on a simplistic model of sagittal plane human walking,(ii)the compliance of the human hip and knee joints were not accounted in the dynamic model, (iii) the fabricated device has inherent friction in the joints, which can be reduced but never completely eliminated, (iv) the torque-deflection curves of torsional springs used in the experiment may not be completely linear.

### Data for a Range of Treadmill Speeds

**Figure 9:**
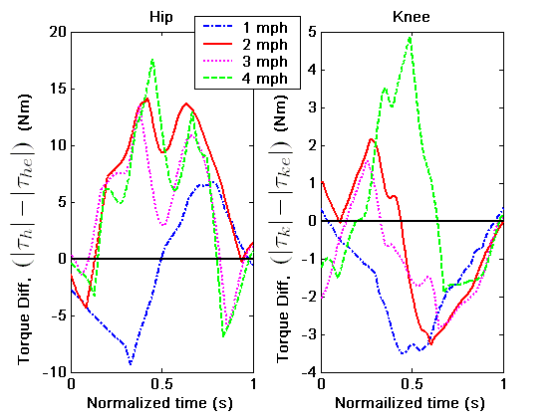
**Joint torques comparison with and without the device**. The difference in hip and knee joint torques without and with the exoskeleton for treadmill speed variation between 1.0–4.0 mph. Higher the positive area under the curves, the exoskeleton is more effective at that treadmill speed.

**Figure 10:**
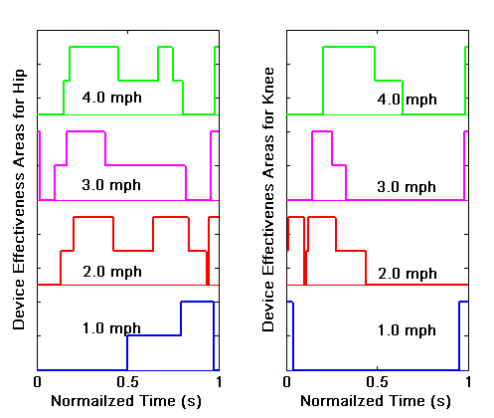
**Device Effectiveness**. Device effectiveness in terms of both 'sign' and 'magnitude' for different treadmill speeds at Hip and Knee joints.

In order to evaluate the robustness of the design to variations in treadmill speed, the joint motion and interface force-torque data was collected for a range of speeds between 1.0 mph – 4.0 mph. Figure 9 shows the difference between the absolute magnitudes of torque required in (ii) and (i), i.e., without and with the exoskeleton, for treadmill speeds of 1 mph – 4 mph. This quantity is labeled as (|*τ*_*h*_| - |*τ*_*he*_|) for the hip and (|*τ*_*k*_| - |*τ*_*ke*_|) for knee. In these comparisons, the time scale was normalized over different treadmill speeds to show the relative effects. In these graphs, the positive area shows the regions of the swing where the device is effective. The larger this area is, more effective the device is at that speed. For the hip joint, we see that the curve corresponding to2 mph treadmill speed has the largest positive area and for the knee joint, the curve with4 mph treadmill speed has the largest positive area. It is possible that further adjustments of the stiffness of the torsion springs may improve the performance even further. Figure 9 focussed on the magnitude of the torque and their sign can be further investigated. For example, the torque in (i) may have the same or opposite sign to (ii). The sign of the torque is more clearly described in Figure 10, where the device effectiveness at different treadmill speeds is compared in terms of sign but not the magnitude. In these figures, a unit step signifies that the device is effective in magnitude but torques in (i) and (ii) have opposite signs. Two steps signify that the device is effective both in magnitude and sign. The larger the area under the curve, the higher the effectiveness of the device at that treadmill speed. On comparison, we see that for the hip, the 2.0 mph treadmill speed trial has the maximum area. The 3.0 and 4.0 mph speed trials also have comparable areas, which shows the robustness of the design over changes in the treadmill speed. For the knee joint, for 1.0 mph, the area under the curve is very minimal – indicating that the knee has poor performance. For other treadmill speeds, the area under the curve is not as small as that of 1.0 mph – indicating neither a good nor a poor performance.

## Conclusion

In this paper, we presented a simple un-motorized bilateral exoskeleton for swing assistance and training of motor impaired patients. This exoskeleton is aimed at reducing the physical and financial costs associated with therapist assisted training. The device consists of two segments – thigh and shank with torsion springs at hip and knee joints. Stiffness of the springs and their equilibrium configurations were the design parameters, which were optimized based on the required performance of the exoskeleton. We modeled the human leg with two links, thigh and shank segments, moving in the sagittal plane. The foot was modeled as a point mass and the hip had the motion of an inverted pendulum. The dynamics are developed when the device is strapped to the leg. In the simulation, we observed that the device helps the leg during swing to clear the ground and go to a desired final configuration. We also performed simulations with change in leg mass to evaluate the robustness of the design to variation of system parameters. We found that the system was robust for up to 50% change in leg mass.

An exoskeleton was fabricated based on the optimized parameters from simulations. This device was tested on a healthy subject at different treadmill speeds. To show the effectiveness of the device, we compare two different cases. In case 1, we estimated the torque applied by the human joints when walking with the device using the joint kinematic data and interface force-torque sensors. In case 2, we calculated the required torque to perform a similar gait only using the kinematic data collected from joint motion sensors. On analysis, we found that at 2.0 mph, the device was effective in reducing the maximum hip torque requirement and the knee joint during the beginning of the swing. These behaviors were retained as the treadmill speed was changed between 1–4 mph. These results were remarkable considering the simplicity of the dynamic model, model uncertainty, non-ideal spring behavior, and friction in the joints. We believe that the results can be further improved in the future. Nevertheless, this promises to provide a useful and effective methodolgy for design of un-motorized exoskeletons to assist and train swing of motor-impaired patients.

## Competing interests

Authors applied for a patent relating to the content of the manuscript. University of Delaware holds the rights to the patent.

## Authors' contributions

KKM performed simulations and detailed exoskeleton design. SKB helped in subject testing of the exoskeleton. SKA provided overall guidance to the project including conceptual design, dynamic simulations, and data interpretation. All authors read and approved the final manuscript.
